# The Family Doctor

**Published:** 1905-12

**Authors:** John Dacre


"*Scire est nescire, nisi id me*
*Scire alius sciret.*"

DECEMBER, 1905.

THROUGH

### THE FAMILY DOCTOR.

The Presidential Address, delivered on October 11th, 1905, at the opening of the Thirty-second Session of the Bristol Medico-Chirurgical Society.

BY

JOHN DACRE, M.R.C.S.

It was in March, 1874, that this Society first saw the light, under the presidency of the late Dr. Brittan, and from its infancy, through childhood and adolescence, up to the present, the thirty-second year of its sturdy life, this chair has been occupied in sequence year by year by men who have emulated his good example and have maintained the dignity of the office at a high level.

I therefore feel very deeply the position in which I find myself, and before proceeding further I beg to tender to you my hearty thanks for the honour you have conferred upon me. I assure you that to the best of my ability I will perform my duty, and I only ask for your kind co-operation in guiding the business of the coming session. So large a number of my predecessors have been men holding high rank in our own immediate medical world, that I feel in electing me you have paid a great compliment to the general practitioners of this city—a large and, I venture to say, wholesome brotherhood to which I am proud to belong, and for which, during this year at any rate, it is my pleasure and my privilege to be the representative.

And now after expressions of gratitude must come a humble confession—a confession of weakness. For months past the compilation of my inaugural address to you has been a source of great anxiety. I anticipated the difficulty, but I had not the slightest conception that the difficulty would be so great. It was not that the choice of a subject gave trouble—that was bad enough—but the setting down of one's thoughts in appropriate language has proved a laborious trial. I have often wished that for this one year, at any rate, I belonged to the class that are born to greatness, but it has been brought home painfully to me "that greatness has been thrust upon me." Men with a gift to write can take a theme, however humble, and with a fluent pen adorn it, but such a gift is not to me. So with this somewhat cheerless preamble I can only beg that you will be lenient with me, as with halting steps I trespass upon your time for a few moments in laying before you a few views that have been forced upon me during the eighteen years that I have been engaged in family practice.

Although still young in years, I feel that professionally I am sufficiently ancient to plead justification for taking such a course, for it is now twenty-seven years this very week since I first joined the ranks in medicine. It is impossible for a man to be engaged so long in our arduous calling, if he be at all thoughtful, without his formulating *some* ideas. From force of circumstances his notions may possibly be, and no doubt are, often fashioned in a narrow groove; but however narrow and however insignificant they may be, it can do no possible harm to let them see the light of day. The calls upon the energy and the time of a family doctor are so great, that there is little margin left for the development of those qualities which would be instrumental in adding to the store of general medical knowledge. It is a thousand pities that this is so; for the opportunities for collecting clinical material of value in general practice are so great, that I for one would gladly welcome any method whereby such material, often lost, could be utilised.

Dr. Michell Clarke, in the very charming address which he delivered in June last before the local branch of the British Medical Association, said very wisely: "After all, a physician, however erudite he may be in other branches of knowledge, should be learned as a physician first and erudite in other ways afterwards." With this I absolutely agree; but I fear that it is this very consciousness of weakness that keeps our family doctors from coming forward more often than they do at our medical meetings. They feel that unless they can bring to bear upon their efforts that erudition which more gifted men find no difficulty in displaying, that possibly their offerings will not be well received. But in this year of grace let us do our best to prove that this is an erroneous notion. Please let me beg of you to come forward with your plain, unvarnished tale, and I give you a pledge that your mite will be welcome. If in the fierce light that beats upon discussion you should feel aggrieved, let me advise you that it will be all in a brotherly spirit; therefore be not discouraged, for the success of a paper is measured by the discussion it provokes, and far better than a chilly silence is a perfect storm of opposition. It would afford me the greatest personal satisfaction during my year of office to see the names of my fellow general practitioners more often in evidence on our monthly programmes than has been the case in the past. If this is asking too much, I would at least add an earnest invitation to them to be present at our meetings in greater numbers, and allow themselves to be tempted to join in our discussions. In hospital life there is no safer guarantee of efficient, up-to-date work by the staff than the presence of students, and I feel sure that in this post-graduate gathering of ours there can be no better stimulus to good work all round than the constant presence of a large and varied audience. It is always a great pleasure to even listen to an animated discussion, and though one side or the other may carry off the palm, still like good sportsmen on the tennis lawn or the cricket field the vanquished take their beating well. We meet in this room on common ground, and in the most friendly spirit criticism is given and received. It is a gymnasium where each one may join in the fencing if he so desires; but however sharp the bouts may be, we know with a certainty that there are no poisoned rapiers in our rack, and our foils are always buttoned. If, therefore, in the few moments at my disposal I can by any words of mine suggest a thought that will be of service in bringing into line the hidden talents in the minds of our general practitioners, or encouraging an effort to overcome the restraining influence of modesty, I shall feel that I have not spoken in vain.

In the early days of my work I frequently heard words spoken in disparagement of the family practitioner, and possibly I was guilty of joining in the conversation myself. He was often held up to ridicule and contempt as a man of inferior clay and poor attainments, whose very name was synonymous with incompetence. He was spoken of as a man holding a position to be shunned by all who (according to their own standard) were men of a higher sphere of intellect. He was dubbed with the ill-sounding sobriquet of G. P., and, like his namesake amongst his patients, he was so far a general paralytic that time alone would account for his effacement, and in his place would arise a set of superior persons who would condescend to attend to the ailments of poor suffering humanity at high fees and by appointment only. Experience has taught me to smile at this puerile nonsense. I have become a family practitioner myself, and though my remarks may savour of egotism, I have come to the conclusion that he is not such a bad fellow after all.

The general public in recent years has become so much more enlightened in matters medical that it has become more imperative than formerly for the men who wish to be successful to set before themselves a very high ideal of excellence. The layman has come to discuss medical topics with a familiarity that is often astonishing. He knows all about germs, will talk glibly of appendicitis and of adenoids, and he is just as much at home in discussing the theory of the causation of ague as he is in speaking of the advantages and disadvantages of the open-air treatment of phthisis. He understands the baneful influence of uric acid in his system, and he is quite prepared, without any prompting even, to criticise our prescriptions. Whether this enlightenment adds to his own material happiness is doubtful, but that it exists there is no doubt whatever, and we must recognise it. In days gone by "a shake of the head and a vague allusion to the liver" might suffice, but to-day that is insufficient. Our patients now require "chapter and verse" for all our assertions, and happy the man who is always in a position to give them. Under these altered circumstances, in order to preserve our self-respect and our superiority, it becomes more and more of a necessity that our medical education should be standardised to meet the modern requirements; the young man of to-day must be made to recognise that when his time comes he will have to face a public whose questionings will be far more rigorous and searching than those of the examiners, whom he fears, at the Conjoint Board. He may fondly imagine that when he has crossed the Rubicon of his final examination and is legally entitled to write certain magic letters after his name that all will be plain sailing, but he will find that it is not so.

We know as a fact that the majority—perhaps it would be better to say nearly the whole—of the students of medicine will eventually have to earn their living as general practitioners. Whatever their hopes and aspirations may be in the beginning, or whatever exalted ideas they may develop in the course of their training, there will come a time when the battle of life must be faced, and they will find that the only courses open to them are those that lead to general practice. They will find that in the keen competition which has already begun—even in the student days—the higher walks of medical life will be won by those who have a right to them, and the medical student as we know him is not a fellow to be discouraged because he is beaten in the race by a better man. To borrow a Napoleonic metaphor, every student starts with a field marshal's baton in his knapsack, but he knows, without the telling, that precious few will ever wield it, and the rest must be contented with a position that carries a lower title; but I will say this, and I wish to emphasise the point, that though the position may be shorn of the patent of nobility, still it is no less honourable in the opinion of his fellow-officers and in the eyes of the world at large. The spurs that are to be won by the general practitioners of medicine are just as bright as those that belong by right to our consulting physicians and our operating surgeons. They can be honestly won by honest work, and though they may not be of gold, yet steel is very useful, and at any rate we have the proud satisfaction of feeling that the burnish is not electro-plate. By accident, or good luck, whichever term you put to it, these spurs may be won in early life, but in most cases they only come after years of arduous toil. How can we ensure that a still larger number shall attain the full measure of success at an earlier stage? I think this can be done by some improvement in our early education.

Do we give our boys the best possible start? Do we teach them how to earn their living at the earliest possible moment? I do not think so. To be practical, if it is to be the rule that young doctors are not going to earn their first fee before they are 28 or even 30 years of age, then our doors will be closed to all but the sons of the wealthy, and our ranks will lose some good men. In my time I have seen the length of the curriculum altered from a minimum of four years to a minimum of five years; but even this does not suffice, for students are so alarmed at the magnitude of their work that they only venture before the examiners with one subject at a time, with the result that the five years are easily expanded to a length that gives rise to serious misgivings in the minds of parents and guardians. In my humble opinion our young men do not learn their responsibilities early enough. I would myself like to see more of the spirit of the Royal Navy infused into our early medical life. The midshipman may learn in a hard school, but it is good for him and good for the service. He is very quickly made to understand and to realise the responsibilities of his calling, and at a very early stage in his career he becomes a capable and self-reliant individual. He not only knows his work, but he has learnt concurrently how to apply it.

To the earnest student of medicine there is no doubt that the facilities and opportunities for acquiring a reasonable knowledge of his profession are manifold; and it is entirely his own fault if he fails to take advantage of them. But granted that he has made such good use of his time that he becomes a walking encyclopædia, still he will find that when he comes to apply his knowledge to actual medical practice, there is still an enormous amount to be learnt, and instead of the bed of roses that he may have anticipated, he finds that his path is full of thorns. I do not say this with any motive of discouragement, for I know that the difficulties are over-ridden in time; but I should like to see something added to the curriculum to give our students an opportunity of practice in the homes of patients, where they would learn to avoid those pitfalls that chance at almost every step.

I know it is rank heresy to say so, but I cannot help thinking that it was a mistake entirely to do away with the old-fashioned custom of domestic pupilage, especially as nothing was suggested even approximately to take its place. Under the old régime a student, at the same time that he was studying in the hospital and the schools, was quietly, almost unconsciously, assimilating a vast amount of knowledge and material in the private dwellings of patients, under the guidance and supervision of the master, an asset that was instantly of service to him upon his obtaining his qualification and starting on his own account. Now when he enters the profession he finds that he has to learn all this for himself: he has to accommodate himself to the difficulties of managing patients, and many a hard knock he will get before he has thoroughly learnt his lesson. Rebuffs wound more deeply now, for he is a fully qualified medical man, and possibly has acquired an undue sense of his own importance. But he must be prepared to experience much heart-burning and many little trials of temper before he finally feels his feet, and settles down to the practical methods that ensure success. I well remember in my own beginnings having to hear things that were hard to put up with. On one occasion I was making my first call at a house on behalf of my dear old partner Mr. Corbould, who chanced to be away from home. I had evidently been seen as I approached the house, and my somewhat youthful appearance had evidently not impressed the good people, for after I had been waiting in the little sitting-room for a few minutes, the daughter of the house came in, and said: "Mamma sends her compliments to you and thanks you for calling, but she thinks she would rather wait until your papa comes back." This was a sharp knock, but fortunately I took it philosophically. It is so long ago that I hardly remember what I did or said, but before I left that house I had been upstairs and seen the patient, and to this day that family remains on my list. I take no credit to myself for this result: it was simply the outcome of the experience I had gained during my four years' pupilage in Leeds. We boys soon got tired of being sent back—sometimes rather sharply—a second time to a patient who had refused to see us, so we set our wits to work to make one journey suffice.

It would be presumptuous on my part to suggest a remedy for what I honestly feel is a defect, but if it came to a discussion and I were asked to give my views, this is what I should say: In the first place, I am a great believer in the virtues of the office of "student for the week." It is a fine thing for the students in Bristol to know that each and everyone has at any rate at some time in their career been a hospital resident. But I would extend it. I would have a student for the week not only at the Royal Infirmary and General Hospital, but I would have one at the Children's Hospital, at the Fever Hospital, and at the Workhouse Infirmary. I would let each man in his turn take a three months' course as assistant to the medical officers at the Bristol Dispensary. I would appoint a certain number of general practitioners of repute on the teaching staff, and for the last year of the curriculum I would have students become their resident pupils and assist them in their work. Further than this, I would suggest that some general practitioner of medicine be put upon the Court of Examiners. In this way it might be possible to evolve a man who not only knows his work, but who knows how to go about his business.

It is hardly necessary for me to say that a young doctor does not jump into a large practice all at once; but sooner or later, when success has smiled upon him, he will find that in order to keep pace with his work he must contrive some procedure with method in it, and he must combine efficiency with rapidity, or he will fall into the fatal error of superficiality and slovenliness.

The fact that perhaps is most forcibly brought home to the mind of the practitioner in the initial stages is that a very large proportion of the ailments that he is called upon to treat are what in his hospital days he would have considered trivial—so trivial indeed as to be absolutely beneath his notice with so much that is serious around him; but he must recognise that upon taking a right line in treating these insignificant cases much of his ultimate success as a doctor will depend. He will be compelled to learn that what *he* may consider are trivial ailments are not trivial to the patient, and it will be well for him to formulate a method of placing himself in his patient's shoes and saying to himself, "What if I felt as this man does; should I consider it trivial?" In fact, it requires no great effort to recall to our recollection the fuss we ourselves made over that last toothache, or the magnitude of our woe during that temporary attack of dyspepsia. It is well to recollect these things, for "a fellow-feeling makes us wondrous kind," and the poor sufferer is already half relieved when he feels that he is pouring his doleful tale into a sympathetic ear.

A patient does not take the trouble to consult a doctor (especially when he is prepared to pay for the privilege) without anticipating that he is to receive some consolation in his suffering and a word of advice to give him relief. He may have absolutely nothing physically wrong with him — from top to toe his organs may be as sound as the traditional bell, but there is a rift within the lute—a trivial rift maybe—but it is sufficient to put all things out of tune. It would be cruel and brutal to give him scant attention and send him away brusquely. No! Far better to devote some time to him and give him a thorough examination. In his morbid state of mind there is no doubt a secret fear, which he has not the courage to express, that his symptoms may possibly be the beginnings of some grave organic disease. With the irritating cough of pharyngeal catarrh he imagines that he may have consumption. How can you expect to convince him that his lungs are sound if you have not examined his chest? The palpitation that arises from flatulent dyspepsia compels him to fancy that he is suffering from heart disease. How can you convince him to the contrary if you have not used the stethoscope? That vague pain in the back makes him fear that there is something seriously wrong with his kidneys. How can you negative this to his complete satisfaction if you never examine the urine? When all this is done, and not till then, are you master of the situation, and able with conviction and satisfaction to the patient to express an opinion on his case. You can then say truthfully, "I have examined you very thoroughly, and I find absolutely no trace of any organic disease about you. Your symptoms are due to so-and-so, and I think it will be no difficult matter soon to set you right." It is a happy moment when a patient says, "Good-bye, doctor; I thank you for your attention. You have taken a lot of pains with me. I thought when I came to you it was a bad look-out, but I feel better already." In citing this example, I do not wish my words to be misconstrued into an advocacy for unduly magnifying minor ailments, for that would savour of humbug. My desire is rather to establish a principle of thoroughness in the examination of all cases, however unimportant they may appear at the time to be. It is only by thoroughness of examination that we can put ourselves in a position to give our best advice, and it is only by honest painstaking that we can hope to avoid falling into error. It should be a guiding principle to us all to do our very best for the patients who entrust themselves to our care, and it is irrational to suppose that success will attend our efforts if we do not at first find out what is the matter with them.

This leads me to my next point, the absolute importance of diagnosis. Diagnosis is the only sound foundation of successful therapeusis, the very bed-rock of rational and scientific treatment. Without it we are simply treating symptoms, and become nothing more nor less than empirical drug dispensers. To discover the *fons et origo mali* whenever possible should be a first principle in our practice, and although we may find this is a great difficulty, in fact at times an absolute impossibility, still there will be few cases where it will be impossible to arrive at any rate at a working hypothesis, and we need not be discouraged in our honest efforts to unravel the problem. With this idea before him, the family practitioner, with his time so fully occupied, must cultivate a method of rapidity, sifting evidence and seizing upon essentials, and it is surprising how he gets unconsciously into the habit of rapidly interpreting facts that are disclosed to his examination. From the very nature of his work it is not absolutely necessary that he should come to a complete judgment at the first visit, but at any rate he must so far grasp the salient points as to place his patient in a position of safety until he sees him next time. It is here perhaps that the general practitioner holds a considerable advantage over the man who is a pure consultant, for his opinion need not necessarily be formulated until he has had further opportunities of following up the sequence of events, whereas the consultant is merely able to give an expression of opinion and a guide to treatment on the evidence that is brought to his notice at one visit only.

Prof. Clifford Allbutt in one of his many addresses has said: "This training of sense and inference in the absence of science made Hippocrates a physician of no mean skill and insight. It has made a valuable helper of many a doctor of modest scientific acquirements, and it makes perhaps the larger part of the gifts of the best of us, that quality without which all the rest is vain." These are happy words to encourage us to train our faculties of observation. Let us see that the hand and eye be true. Instruments of precision are of inestimable value, but sometimes I feel it brought strongly home to me that too much reliance is coming to be placed upon them, and primitive methods may become neglected. I do not like the idea of diagnosis by machinery coming to the forefront of our calculations. I would rather trust to the utility of the senses, and only make use of instruments of precision to corroborate what one has already conjectured.

It would be well if we endeavoured more often to develop the attributes of the scout and to study more closely the methods of woodcraft. By woodcraft I mean the tracking of animals in a state of nature in the ordinary pursuit of game or of hunting, and perhaps no better example could we give than that which occurs in our adjoining county of Somerset in connection with the Devon and Somerset Staghounds. If the hunting of the stag were conducted on the lines that are followed in the shires in following the fox, successful days would be few and far between. Deer, as we all know, run in herds, and to send a whole pack of hounds into a covert would result in the pack being divided, so that a couple would follow one deer, three or four would follow another, and the whole hunt would be in confusion. It is necessary, in order to pick up a warrantable stag, that his whereabouts should be located, and this duty devolves on an individual who is called the harbourer. He is a man skilled in the ways of the deer, and who, by natural instinct and years of practice, has developed a wonderful power of tracking. His object is to find a stag old enough to be worthy of hunting, and for some days before the meet he makes it his business to search for evidence of an old stag being in the neighbourhood. Young ash trees must be examined to see if the young shoots have been nibbled. The bark of trees is scrutinised to find a transverse bite rather than the up-and-down gnawing of the hinds—the stems of trees show signs of having been rubbed whilst fraying the antlers—and from the height of the injury he can judge of the size of the animal that has caused it. But most important of all, he must look for the footprints, the slots as they are called. These he will find on the soft soil at the edge of a pool where the stag takes his bath before retiring to his rest, and also at some gap or other leading into the covert. I need not describe the individual peculiarities of the slot of an old stag; suffice it to say that they are so striking as to make them unmistakable to the practised eye of the harbourer.

With these preliminary observations he has made himself acquainted, roughly, with the usual behaviour of the deer in this particular district, and on the afternoon before the meet he takes a run round in order to obliterate any tracks that are to be seen, so that he starts with a clean slate on the eventful morning, when he must be up with the dawn on the trail of the deer as early as possible after the midnight feeding, for he knows that they retire to lie down for the day at the first glint of daylight. He casts around until he finds the particular slot that he wants, and he must find impressions of it at an entrance to a covert to show that the animal has gone *in;* and more than this, he must go over all the gaps in the fence to make certain that there are no impressions which show that he has gone *out.* When he is satisfied with all these points he knows that the stag has made his bed in that particular plantation; he is said to have harboured the animal; one may say that he has diagnosed—stag. For the sake of his own reputation and the success of the hunt he must be quite certain about the result. It will not do for him to say, "I think there is a stag somewhere about." No, he must be in a position to say that the animal they want is there, and it is great testimony to his method and to his skill when I tell you that he rarely, if ever, makes a mistake. He tells the huntsman at the meet the result of his search, and then by means of a few hounds called "tufters" the warrantable stag is roused from his lair, the rest of the pack is laid on, and then begins a chase which has not its equal in any other part of the British Isles.

In a very suggestive paper written by Sir Lauder Brunton many years ago on the method of Zadig in medicine he brings into prominence these faculties of tracking, and as his words describe them more eloquently than I can do, I may be pardoned for the quotation. He said: "It is not only the outward signs that we have to differentiate. We are obliged to judge of the unseen from the seen, and from the objective data that are presented to our senses we must draw conclusions regarding the processes which are going on or have already gone on in the persons of our patients. We must, in fact, track symptoms which we see back to their causes. Now the process of tracking appears to be a fundamental one in man, and one which really seems to be a remnant of the qualities possessed by our prehistoric ancestors. It is a reversion in the faculties of the mind; and the keen interest in tracking game by which prehistoric man was enabled to exist still evidences itself in the intense eagerness with which boys read in Cooper's novels about Indians following the trail, or adults will pore over narrations of Gaboriau's stories of the police on the track of criminals.. . . . There can be little doubt that the profession of medicine is a most intensely interesting one, and I am inclined to think that its special charm is to some extent at least due to the fact that it is constantly demanding the exercise of these qualities of tracking which we find in Cooper's Indian heroes."

As described by Voltaire, "Zadig was a young man who, disgusted with life, retired from Babylon to a lonely place on the banks of the Euphrates, and there studied animals and plants until he saw a thousand differences where others could only see uniformity. One day one of the queen's eunuchs, followed by a band of officials, came hastening past, and asked Zadig, 'Have you seen the queen's dog?' Zadig modestly answered, 'A bitch, I think, not a dog.' 'Quite right,' said the eunuch, and Zadig continued, 'A very small spaniel, has lately had pups, limps with the left foreleg, and has very long ears.' 'You have seen her, then,' said the eunuch. 'No!' said Zadig, 'I have never seen her, and did not even know that the queen had a dog at all.' Asked to explain, he said, 'I noticed one day in the sand the tracks of an animal which I easily recognised as those of a small dog, long and evenly marked furrows imprinted where the sand was slightly raised between the footprints told me it was a bitch, whose dugs were drooping, and that consequently she must have given birth to young ones only a few days before. Other marks of a different character, showing that the surface of the sand had been constantly grazed on either side of the front paws, informed me that she had very long ears, and as I observed that the sand was always less deeply indented by one paw than by the other three, I gathered that the bitch belonging to our august queen was a little lame, if I may venture to say so.'"

This may all present the glamour of a fairy story, but I think that perhaps it would be as well if we brought to bear, more often than we do, in our efforts in arriving at a proper diagnosis, the subtle and unerring methods of the scout, the harbourer, and of Zadig.

In addition to this I would plead for a cultivation of the artistic sense, that perfect one which grasps the ideas of symmetry and proportion. I think it may be accepted as a truism that the germ of this sense lies latent in the minds of most men, but in doctors I consider that every encouragement should be given to its development. This sense should be cultivated to such a degree, that it not only appreciates that which is perfect, but should also feel grieved at the sight of anything false. It finds expression in different men in different ways. In one it may be an appreciation of form and colour, in a second it may rapidly discern the various points of a good horse, whilst a third will find his sense gratified by looking on the beautiful tower of All Saints, Bristol, as he walks up Clare Street on a sunny afternoon. The doctor, on the other hand, should have a vivid mental conception of the ideal human form, so that any deviation from the normal, in one way or another, should instantly offend his artistic sense and as rapidly prove to him that some abnormality is present.

Perhaps more often than we are aware do we carry out the elucidation of our cases by building up a living picture. Facts that are on the surface are registered by the sensitive retina unless one is afflicted with intellectual blindness, but there are things lying hidden within that require the use of other senses to bring them out. We may have to call into use our sense of hearing, our sense of smell, and perhaps not the least important the sense of practised palpation—the sense which gives us an eye at the end of every finger, and which in its perfect form we call *tactus eruditus*. We may even be called upon to utilise the faculty of imagination, the gift which in the words of Shakespeare "bodies forth the form of things unknown, turns them to shapes, and gives to airy nothings a local habitation and a name." Using our senses, one almost might say, like the lenses of a stereoscopic camera, we gradually build up a picture until it more and more accords with the clinical symptoms—the details more accurate, each and every point standing out more and more prominently in relief, until at last, clearly defined before us in all its completeness of detail, stands our clinical picture—our diagnosis. Until this picture is complete in all its details we must not rest satisfied, and it is when we are baffled in the attempt that it is wise to call upon a brother artist to come along and help us in the matter. And here it is, I think, that the value of a consultation can be so satisfactorily explained in this pictorial fashion. The practitioner relates his experience of the case; he gives the symptoms, the progress, and its daily variation; in fact, he places the picture so far as he is able to portray it boldly upon the easel. He says, "So far I have got, but beyond this point, as you will see, the picture is not perfect, and I want you to complete it for me if you can." What a much better chance there will be of the master hand making a brilliant drawing under these circumstances than if he had not had before him the helpful and preliminary sketch!

In speaking of the careful compilation of this clinical picture, it may be gathered that I am opposed to the sharp practice of snap-shot diagnosis, which in the words of the cautious Scotchman may be said to be "varra smart, but it's varra risky." Only once did I ever allow myself to be tempted into a diagnosis of this kind—a diagnosis on true Sherlock Holmes lines. The gardener of a patient of mine came to see me suffering from an irritable rash on the hands and forearms. On questioning him about his work, I found that he had recently been busy in repotting a large number of plants of *Primula obconica*, which evidently was the cause of his trouble. The very next patient that came into my room was a lady, a stranger to me. She unfastened her gloves, and laying her hands upon the table, asked me to tell her "what was the matter with her skin." Her hands were an exact reproduction of those of the gardener. The temptation was too great to be resisted. I said, "You are too fond of gardening. You have recently purchased some *Primula obconica*, and you have been busy handling them and placing them in your greenhouse." She gave a little gasp, and said, "You are quite right, but how on earth you know quite beats me." I felt at the time that the pleasure I derived was hardly an honest one, but that it was very much akin to the delight of a conjurer when he sees the bewilderment on the faces of an astonished audience. Of course I had to explain that the patient that had just left the room was suffering from exactly the same thing.

Rather than this clever dramatic method I would encourage a careful formation of clinical pictures in daily work. In a long course of years engaged in family practice what a wonderful collection of pictures, we might call them "cerebrographs," will be gathered together. They make an asset which cannot be appraised at a price and which cannot be taken away. They are stored away in that gallery of magic which we call memory—a huge reference library that we, and we alone, can utilise for the benefit of our clients. This is really what our patients call "knowing their constitution." Now here is a phrase that is usually used in terms of cynicism, not to say flippancy, but I am firmly convinced in my own mind that it is a matter not lightly to be disposed of. Surely it is of some practical value that the medical man should be conversant with the thousand and one frailties that his experience has taught him beset their path, and it is certainly within the recollection of each of us that the knowledge of the past history of the individuals of the families entrusted to our care has many a time proved of absolute practical value. With these pictures stored away in the pigeon-holes of our memory, it is far easier to call upon them for reference than to refer to the laborious and arid notes that lie hidden away at home in a book that is possibly unindexed. It is surprising how they come back like a flash when occasion calls, and how they can be brought into requisition when we most require them. Not only are there the pictures of our successes, but there are the pictures of our mistakes, and we all make them sometimes, for it has been well said "the man who never made a mistake never made anything," and I believe it. There they are, these grim memories, and it does no harm to have a look at them sometimes. They are not difficult to find; there is no need to shake the leaves to find them, or to search in the pockets of the mental portfolio, for they only too readily show themselves; like a well-thumbed page of a book they are disclosed without any trouble. Framed in black and engraved in no uncertain lines, bitten in with the acid of remorse, they stand out clear and vivid as a vigorous etching would--and not only do they warn us against similar pitfalls and mistakes, but in their contemplation we are chastened and more conscious of that proper sense of humility which compels a lenient view of the errors of our brethren.

In producing these living pictures, I do not wish you to approach the matter as a visionary, a dreamer of idle dreams, or even as an impressionist, but rather as a disciple of the realistic school with a slavish attention to detail, endeavouring with every sense to detect the slightest flaw, probing, digging, looking, leaving not a stone unturned until with conviction brought firmly to the mind that the track is struck at last, you pass to the full attainment of your quest, and in spite of every difficulty, and in the face of every natural obstacle, you have realised the solution of the problem which is before you.

I fear that the views I have brought forward have displayed me in a somewhat fantastic light, but there is charm in fantasy to my mind. I have endeavoured to revive your interest in a method whereby our daily task, instead of being a sordid, dry-as-dust ordeal can be made a delight; instead of living laborious days, if only we can cultivate an artistic enthusiasm in our cases, our work will cease to be labour and our vacations will become to be reckoned as only another mode of enjoyment. It has often been said that we scorn delights. I repudiate the statement stoutly. No one welcomes and appreciates delights more than the hard-worked doctor, and with this artistic method which I have endeavoured to set before you, I venture to think that the "laborious days" are less in number than most of us suppose.

And now for a practical suggestion. Each one of you, my brother practitioners, possesses a gallery of art at which we should like to have a peep. May I beg that you will allow the best of your pictures to be shown in our exhibition? I you are skilled in draughtsmanship you will place them on our walls; failing this you may draw word pictures, which I feel certain we shall appreciate, and which will add to the mutual enlightenment for which our Society exists.

I have endeavoured to set forth, I fear most inadequately, the idealistic side of family medical practice, for it is better in times like these to take pleasure in the brighter side than to recount the pains and vicissitudes of which we are only too conscious. The appreciation of our work is often felt, but perhaps is seldom voiced; but this is what Robert Louis Stevenson says in his dedication of *Underwoods:* "There are men and classes of men that stand above the common herd—the soldier, the sailor, and the shepherd not infrequently, the artist rarely, rarelier still the clergyman, the physician almost as a rule. He is the flower, such as it is, of our civilisation, and when that stage of man is done with and only remembered to be marvelled at in history he will be thought to have shared as little as any in the defects of the period and most notably exhibited the virtue of the rest. Generosity he has such as is possible to those who practise an art, never to those who drive a trade. Discretion tempered by a hundred secrets, tact tried by a thousand embarrassments, and, what are more important, Heraclean cheerfulness and courage. So it is that he brings air and cheer into the sickroom, and often enough, though not so often as he wishes, brings healing."

Our dear old friend, Oliver Wendell Holmes, in his poem "The Morning Visit," gives words of advice that sound pleasant to our ears and give us encouragement in our task. He says:—

"And last, not least, in each perplexing case
Learn the sweet magic of a cheerful face,
Not always smiling, but at least serene,
When grief and anguish cloud the anxious scene.
Each look, each movement, every word and tone,
Should tell your patient you are all his own;
Not the mere artist purchased to attend,
But the warm, ready, self-forgetting friend,
Whose genial visit in itself combines
The best of cordials, tonics, anodynes."

With words like these ringing in our ears let us take courage, and be assured that whatever we do let us do it with all our might, and ever keep before us the watchword to do our duty to our patients. They look to us for succour and for help in time of need, for relief in pain, and for sympathy in their suffering. They feel that not only do we stand by them in times of serious distress, but we have always a willing ear for all the little ills that make life miserable. Even more than this, our advice may often be sought in matters not strictly medical; for in times of family trouble there is perhaps no one better calculated to give sound, homely advice than the doctor. He shares their joys just as much as he shares their sorrows. They call him in their gratitude their guide, philosopher, and friend. He is trusted with a thousand secrets, for they have learnt to feel that he is a man in whom they can place their trust, and words committed to his care will lie deep hidden in his heart, never to be revealed; and it is a momentous and terrible time when they trust to him to be the only bulwark that stands between themselves and the dread messenger that comes to all sooner or later.

It is a goodly heritage that has fallen to our lot, and with such a trust as this it behoves us to make it secure that the men who hold such grave responsibilities should be men of the highest integrity and men of the highest efficiency in the best sense of the word, and I here register a hope that some of the giants of our profession and the admirable Crichtons of our craft will ever be found in the ranks of family doctors.

